# The statistical fragility of the management options for reverse shoulder arthroplasty: a systematic review of randomized control trial with fragility analysis

**DOI:** 10.1016/j.xrrt.2023.03.002

**Published:** 2023-04-07

**Authors:** Tom R. Doyle, Eoghan T. Hurley, Martin S. Davey, Christopher Klifto, Hannan Mullett

**Affiliations:** a Sports Surgery Clinic, Santry, Dublin, Ireland; b Galway University Department of Surgery, Galway, Ireland; c Department of Orthopaedics Duke University, Durham, NC, USA

**Keywords:** Shoulder arthroplasty, Reverse shoulder arthroplasty, Randomized control trial, Fragility index, Fragility analysis, Systematic review

## Abstract

Reverse shoulder arthroplasty (RSA) is used in the treatment of traumatic and arthritic pathologies, with expanding clinical indications and as a result there has been an increase in clinical research on the topic. The purpose of this study was to examine the statistical fragility of randomized control trials (RCTs) reporting outcomes from RSA. A systematic search was undertaken to find RCTs investigating RSA. The Fragility Index (FI) was calculated using Fisher’s exact test, by sequentially altering the number of events until there was a reversal of significance. The Fragility Quotient (FQ) was calculated by dividing the FI by the trial population. Each trial was assigned an overall FI and FQ calculated as the median result of its reported findings. Overall, 19 RCTs warranted inclusion in the review, representing 1146 patients, of which 41.2% were male, with a mean age of 74.2 ± 4.3 years and mean follow-up of 22.1 ± 9.9 months. The median RCT population was 59, with a median of 9 patients lost to follow-up. The median FI was 4.5, and median FQ was 0.083, indicating more patients did not complete the trial than the number of outcomes which would have to change to reverse the finding of significance. This review found that the RCT evidence for RSA management may be vulnerable to statistical fragility, with a handful of events required to reverse a finding of significance.

Reverse shoulder arthroplasty (RSA) is used in the treatment of traumatic and arthritic pathologies. RSA was developed in the 1970s to address poor outcomes associated with anatomic shoulder arthroplasty and shoulder hemiarthroplasty arthroplasty in managing rotator cuff deficient shoulders. When reversing the anatomic position of the articulating glenoid and humeral head, it was hoped that by maximizing deltoid function it would lead to improved range of motion and strength, while limiting the risk of dislocation.^14^ RSA case volume has been increasing and between 2011 and 2017 there was an almost 200% increase in the number of RSA being performed in the United States, with an annual incidence of 20/100,000 persons.^28^^,^^46^ This trend is being replicated across the developed world and is expected to continue over the coming decades with growth in shoulder arthroplasty far outstripping that of hip and knee arthroplasty.^25^^,^^46^

Evidence-based medicine has become imperative to safe and effective clinical decision-making since the concept was introduced by Cochrane.^5^ The randomized control trial (RCT) forms level I evidence at the top of the pyramid of evidence.^3^ Orthopedics is a challenging area of medicine to ensure high-quality evidence is available due to often small sample sizes, difficulty in blinding, and patient rejection of randomization.^27^ This is borne out in reviews of orthopedic evidence which have found serious issues with methodological and statistical rigor.^32^ More new topics such as RSA due to the limited published evidence are at the greatest risk of suffering from an underdeveloped evidence base.

To the authors’ knowledge, the use of Fragility Index (FI) and Fragility Quotient (FQ) statistical analysis has not been applied to RCT level I evidence assessing RSA. The FI is a minimum number of events which must be reversed to change the significance finding for a given outcome, while the FQ expresses fragility relative to the size of the trial population. The purpose of this study was to examine the statistical fragility of RCTs reporting outcomes from RSA. Our hypothesis was that included studies would be consistently fragile to a reversal of their stated findings and that the FI would be comparable to the number lost to follow-up (LTFU).

## Materials and methods

### Search strategy

In reference to Preferred Reporting Items for Systematic Reviews and Meta-Analyses guidelines, 2 independent reviewers (T.D. and E.H.) performed a systematic review of the literature in August 2022, including 2 databases (PubMed and Embase).^29^ The search terms used were “Arthroplasty, Replacement, Shoulder” [Mesh] AND “reverse shoulder arthroplast∗” OR “reverse shoulder replacement” OR “reverse total shoulder arthroplast∗” OR “reverse total prosthetic.” The texts discovered using this search strategy were screened by both independent reviewers, with removal of duplicate studies, followed by application of our eligibility criteria.

### Eligibility criteria

The inclusion criteria were (1) RCTs that investigate the management RSA; (2) reporting dichotomous outcomes and statistical significance; (3) full-text studies, published in the last 20 years; (4) published in peer-reviewed journals; and (5) published in the English language. The exclusion criteria were (1) RCTs without a clear randomization protocol, (2) review articles, (3) studies in vitro, and (4) studies involving animals. In cases of disagreement between the 2 independent authors with regard to a study meeting the inclusion or exclusion criteria, disagreements were to be decided upon by the senior author.

### Assessment of evidence

All included studies were assessed for their reported level of evidence, using The Journal of Shoulder and Elbow Surgery criteria.^22^ The Risk of Bias II (ROB II) tool was used to assess the quality of evidence of the included RCTs.^38^ All studies were assessed for the presence and nature of a statistical power analysis. The latest impact factor of the publication journal was recorded.

### Data extraction

Following application of the predetermined inclusion and exclusion criteria, both reviewers collected information on the following variables from included studies in a password-protected database on Microsoft Excel (Microsoft Corporation, Redmond, WA, USA): (1) year of publication; (2) randomization methods; (3) statistical power analysis (type of analysis and reported power); (4) the primary and secondary outcomes as specified in the trial protocol; (5) length of follow-up (months); (6) number of participants included in each of the treatment arms; (7) mean age of participants (years); (8) sex of participants; (9) number as protocol, number per protocol, and numbers LTFU; (10) the reported significance of each event; and (11) all dichotomous outcomes of relevance. As protocol describes the number of patients in a trial who were randomized to a study arm and received the assigned treatment. Per protocol is hereby defined as the number of patients who complete the trial and remain at the end of the follow-up period.

### Statistical analysis

**Figure 1:**
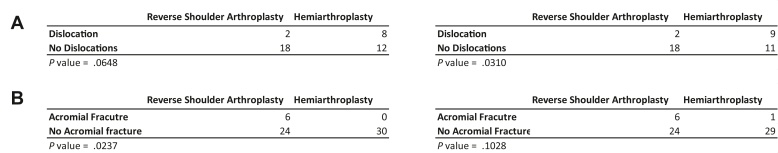
Fragility index calculation, FI = 1 in (**A**) reversal of nonsignificance and (**B**) reversal of significance.

The FI was calculated using GraphPad open source online software (GraphPad, San Diego, CA, USA).^17^ For dichotomous outcomes, both the events and nonevents for each treatment arm were entered into a 2 × 2 grid, and a 2-tailed Fisher’s exact test was used to calculate the *P* value, with α = 0.05. As some *P* values will have been calculated using the Chi-squared test, this is critical. To calculate the FI, the 2 × 2 grid is manipulated until there is a reversal of the original significance finding (Fig. 1). For an outcome reported as significant, it would be manipulated by adding +1 to the events in the treatment arm which had less events, while −1 was removed from the nonevents to maintain the overall population of that treatment arm. This process was repeated until the result became nonsignificant (*P* > .05). Conversely for outcomes which were not significant, the number of events required to decrease *P* to < .05 was calculated by adding +1 to the treatment arm which had more events, and −1 from the nonevents to maintain the population of that treatment arm, and repeated until the result became significant. The number of events changed was recorded as the FI for that outcome. The FI for all outcomes reported in a RCT was calculated in this manner. The median and interquartile range (IQR) of outcomes in a trial was recorded as the overall FI for that RCT. For each finding, the FQ was calculated in Microsoft Excel by dividing the FI by the per-protocol number for that RCT. The overall median FQ and IQR for each study was calculated in the same manner as the FI. We used Pearson’s correlation coefficient when assessing for direct correlation.

## Results

### Literature search

**Figure 2:**
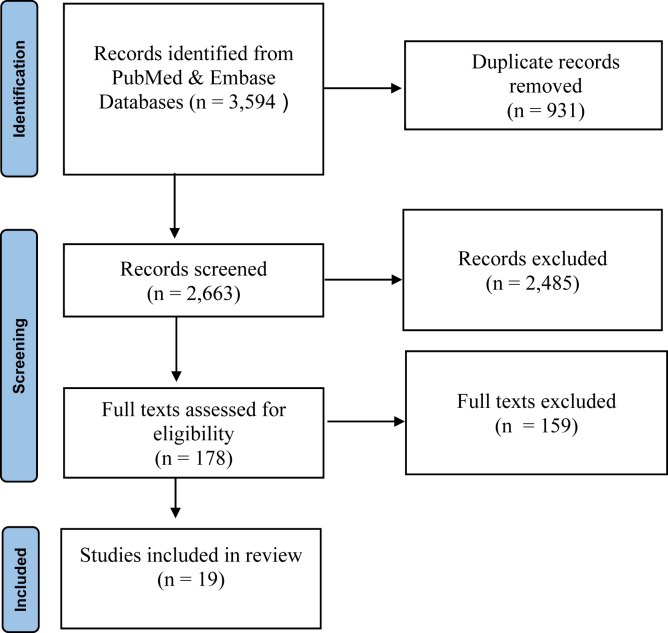
PRISMA flow diagram (Preferred Reporting Items for Systematic Reviews and Meta-Analyses).

Following our initial search, a total of 3594 studies were returned. Following manual removal of duplicate studies, 2663 studies remained for application of our eligibility criteria. Thereafter, the titles and abstracts were evaluated yielding 178 studies for full-text review. Nineteen RCTs met the eligibility criteria warranting inclusion in this systematic review (Fig. 2). The included RCTs represented 1146 patients, with 41.2% being male, a mean age of 74.2 ± 4.3 years, a mean body mass index of 29.9 ± 1.6 kg/m^2^, and a mean follow-up of 22.1 ± 9.9 months.

### Assessment of evidence

The quality of evidence was assessed using the ROB-II tool.^38^ No RCTs were found to be at a high ROB, 13 were found to have a low ROB,^9^^,^^10^^,^^15^^,^^19^^,^^20^^,^^23^^,^^24^^,^^33^^,^^40^^,^^43^, ^44^, ^45^^,^^48^ while for 6 there were some concerns about potential bias^7^^,^^16^^,^^18^^,^^35^^,^^41^^,^^42^ (Supplementary Appendix S1). The current impact factor of the journals in which the included RCTs are published had a mean of 3.6 ± 0.6, with 14 (74%) of the RCTs published in the Journal of Shoulder and Elbow Surgery, 2 in the Journal of Bone and Joint, and 1 each in Journal of Orthopaedic Research, Journal of Orthopaedic Trauma, and Archives of Orthopaedic and Trauma Surgery.

### Fragility index and quotient

From the 19 included RCTs, there were 85 reported dichotomous outcomes. The overall median of FI was 4.5 (IQR, 4-5), and the median FQ was 0.083 (0.065-0.098). The median number of patients LTFU was 9 (range, 3-12). In 13 RCTs (68%), the number LTFU was greater than the median trial FI, while in 6 RCTs it was less than the median FI. A subgroup analysis is shown in Table I. The FI and FQ of these subgroups show that primary outcomes, significant findings, and outcomes where the FI < LTFU were consistently more prone to fragility when compared to the overall median FI and FQ. It also highlights that the majority of reported dichotomous outcomes were secondary and not significant.

**Table I:** Fragility of trial events with outcome breakdown.

Characteristics	Outcomes	Median FI (IQR)	Median FQ (IQR)
All RCTs (n = 19)	85	4.5 (4-5)	0.083 (0.065-0.098)
Reported *P* value			
*P* < .05	11	3 (1.5-7.5)	0.044 (0.028-0.103)
*P* > .05	74	4.75 (4-5)	0.080 (0.602-0.097)
Outcomes			
Primary	7	3 (2-5.5)	0.035 (0.021-0.066)
Secondary	78	5 (4-5)	0.078 (0.053-0.098)
Outcome FI vs. LTFU			
FI > LTFU	29	5 (4-5)	0.097 (0.085-0.129)
FI < LTFU	56	4 (3-5)	0.060 (0.043-0.083)

*FI*, fragility index; *FQ*, fragility quotient; *IQR*, interquartile ranges; *LTFU*, lost to follow-up; *RCT*, randomized control trial.

Data are reported as median and IQR.

### Power analysis

All 19 publications reported a power analysis, with 2 post-hoc analyses^10^^,^^45^ and 17 priori power analyses. The post-hoc group showed greater fragility with an FI of 3.5 (3.25-3.75) when compared to the priori group with a median FI of 5 (4-5). Eleven RCTs (57.9%) were Appropriately Statistically Powered (ASP) meaning they recruited a sufficient sample size to satisfy a requirement of at least 80% power,^9^^,^^10^^,^^16^^,^^19^^,^^20^^,^^24^^,^^33^^,^^35^^,^^40^^,^^43^^,^^48^ while 8 RCTs (42%) were statistically underpowered (SUP) as they did not recruit a population sufficient to achieve 80% power.^7^^,^^15^^,^^18^^,^^23^^,^^41^^,^^42^^,^^44^^,^^45^ The ASP subgroup had a greater median trial FI than the SUP group at 5 (4.50-5) vs. 4 (3.75-4.13). We observed an association between higher powered studies and those with higher FIs, with data shown fully in Table II.

**Table II:** Fragility of trials grouped by power analysis.

Group	RCTs (N)	FI (IQR)	FQ (IQR)
Appropriate Statistical Power	11	5 (4.50-5)	0.083 (0.075-0.098)
>80% power with α = 5%			
Statistically Underpowered	8	4 (3.75-4.13)	0.076 (0.045-0.114)
<80% power with α = 5%			
Priori Power Analysis	17	5 (4-5)	0.083 (0.071-0.100)
Post-Hoc Power Analysis	2	3.5 (3.25-3.75)	0.066 (0.050-0.082)

*N*, number; *FI*, fragility index; *FQ*, fragility quotient; *IQR*, interquartile range; *RCT*, randomized control trial.

There was not a strong relationship between the median FI and the As Protocol (AP) or Per Protocol (PP) population of a trial. The Pearson’s correlation coefficient between AP trial population and median trial FI was R(19) = 0.26, *P* = .256 and between PP trial population and median FI was R(19) = 0.25, *P* = .302. This suggests a weak nonsignificant positive correlation between having more participants and reporting less fragile results. The number of participants LTFU showed a weak nonsignificant positive correlation to the median FI at R(19) = 0.21, *P* = .410. The correlation between publishing journal’s impact factor and median FI was very weakly positive at R(19) = 0.11, *P* = .665. A moderate positive correlation was detected between the AP trial population and participants LTFU which was significant at R(19) = 0.63, *P* < .004. These data are summarized in Table III.

**Table III:** Median trial FI correlation with study characteristics.

Study characteristic	R (FI)	*P* value (FI)
AP trial population	0.26	.256
PP trial population	0.25	.302
LTFU	0.21	.410
Journal impact factor	0.11	.665

*R*, Pearson's correlation coefficient; *FI*, fragility index; *AP*; as protocol; *PP*, per protocol; *LTFU*, lost to follow-up.

## Discussion

The most important finding of this review was that level I RSA clinical evidence was vulnerable to statistical fragility, with a median FI of 4.5 indicating that the reversal of just a handful of outcomes was sufficient to reverse a finding of statistical significance. This should be viewed in the context of the median number of patients LTFU being equal to 9. The median trial lost more patients to follow-up than the number of outcomes which would have to be changed to reverse a finding of significance. These figures add uncertainty to the true validity of a finding of significance, as approximately two-thirds of included events may have had reversed significance findings had there been a more complete follow-up. We cannot know what outcome a patient LTFU had, but it stands to reason that had the trial been completed without their loss, the finding of significance may have been reversed. Events with an FI more than the number LTFU for that trial were more robust than those with an FI less than the number LTFU. These results support the conclusion that a number LTFU > FI is an indicator of potential fragility. Comparative trials of shoulder surgery should consider reporting the FI, FQ, and *P* value for findings to better demonstrate the statistical evidence which informs clinical decision-making.

Almost all published RCTs will report on statistical significance using *P* values, with α = 0.05 arbitrarily set as the cut-off for significance. The *P* value has recently been criticized due to limitations in its clinical relevance.^47^ Due to the small sample size of many RCTs reporting dichotomous outcomes in orthopedics, trials often rely on a small number of events to calculate significance. The FI is a statistical tool first described by Feinstein. For any given outcome, the FI is a minimum number of events which must be reversed to change the significance of the findings using Fisher’s exact test. The FI has no arbitrary point at which it is deemed significant unlike a *P* value and exists independently of the sample size from which it calculated.^13^ A lesser FI indicates a fragile result, while a greater FI indicates a more robust result. The FQ described by Ahmed is produced by dividing the FI by the trial population. This expresses the fragility of the finding relative to the size of the trial, giving added context and allowing for more standardized comparison between trials.^1^

All included RCTs reported a statistical power analysis, which is a positive indicator of statistical rigor in the RSA literature. Of the 19 RCTs, 58% were appropriately statistically powered (ASP) while 42% were SUP. The ASP group displayed more robust results with greater median FI and FQ as seen in Table II. This finding is in keeping with the assumption that well-designed trials will produce more statistically certain results, while underpowered trials will produce more fragile results as they are at risk of type II data errors. There were 17 prior power analysis and 2 post-hoc analysis. The priori analysis is considered to be the most appropriate method to conduct a power analysis, and this convention is supported by the fact this group had a greater median FI and FQ than the post-hoc group.^36^

This review found a nonsignificant weak correlation between both AP and PP trial population and FI. This highlights that the absolute number of participants is not a reliable guide to estimating fragility. The number of participants required will be determined by the size of the clinical effect being measured and its standard deviation. This review found a weak nonsignificant positive correlation between the impact factor of the journal and FI. This highlights that readers should not assume articles are statistical rigorous based solely on the reputation of the publishing journal. Although it should be noted due to the prevalence in this review of articles from a single journal, in this instance this conclusion is limited. There was also a very weak nonsignificant positive correlation between number LTFU and FI, this may be explained by larger RCTs having more patients LTFU in absolute terms and also reporting robust greater FIs.

A fragility analysis in 2018 of the RCTs cited by the American Academy of Orthopaedic Surgeons clinical practice guidelines as “strong evidence” reported a median FI of 2 and a median FQ of 0.022, with 53% of the RCTs statistically underpowered.^4^ While a previously published analysis of 12 surgical fragility analyses found the median FI to be 3 and FQ to be 0.039.^8^ For the purpose of a more focused comparison, we conducted a search for fragility analyses which focus primarily on shoulder surgery. This returned 6 reviews and they report a median FI of 4 (4-4).^6^^,^^12^^,^^26^^,^^30^^,^^31^^,^^34^ These figures suggest that the RSA RCT evidence base is comparable to the wider orthopedic literature, if not mildly more robust. Although it should be noted that in general RSA literature remains fragile, with a small number of events required to result in reversal of statistical significance.

In 2016, the American Statistical Association issued a policy statement confirming that conclusions should not be reached on the basis of whether a *P* value reached a specific arbitrary threshold.^47^ The *P* value does not measure the probability of a true result, the importance of a finding, or the size of an effect. On this basis, the authors endorse triple reporting of *P* values, FI, and FQ as the new standard for RCTs.

### Limitations

One potential limitation of this analysis is the exclusive review of RCTs; this excludes other comparative studies which may have been informative. However, it is the opinion of these authors that fragility analyses should be reserved for RCTs to avoid the risk of selection bias and confounding variables which are sources of fragility found in nonrandomized studies.^2^^,^^39^ A limitation of this review is that it includes fewer RCTs than some other previously published analyses.^8^^,^^11^^,^^21^^,^^37^ However, this is an accurate reflection of RSA evidence pool that is currently available. The primary limitation of fragility analyses is that only dichotomous variables may be included. This led to the exclusion of continuous variables such as the Constant and ADLER scores which are important outcome metrics in shoulder surgery. Such variables cannot be included unless there is a cut-off score which indicates a certain outcome has been achieved, as this then becomes dichotomous data. Another limitation is the high prevalence of included secondary outcomes. Trials are usually powered for the detection of their primary outcomes, and so may be underpowered with regards to secondary outcomes. However, many secondary outcomes are very clinically relevant and so their analysis is both justified and important.

## Conclusion

This review found that the RCT evidence for RSA management may be vulnerable to statistical fragility, with a handful of events required to reverse a finding of significance.

## Disclaimers:

Funding: No funding was disclosed by the authors.

Conflicts of interest: The authors, their immediate families, and any research foundation with which they are affiliated have not received any financial payments or other benefits from any commercial entity related to the subject of this article.

## References

[1] Ahmed W., Fowler R.A., McCredie V.A. (2016). Does sample size matter when interpreting the fragility index?. Crit Care Med.

[2] Andrade C. (2020). The use and limitations of the fragility index in the interpretation of clinical trial findings. J Clin Psychiatry.

[3] Burns P.B., Rohrich R.J., Chung K.C. (2011). The levels of evidence and their role in evidence-based medicine. Plast Reconstr Surg.

[4] Checketts J.X., Scott J.T., Meyer C., Horn J., Jones J., Vassar M. (2018). The robustness of trials that guide evidence-based orthopaedic surgery. J Bone Joint Surg Am.

[5] Cochrane A.L (1972).

[6] Davey M.S., Hurley E.T., Doyle T.R., Dashti H., Gaafar M., Mullett H. (2022). The fragility index of statistically significant findings from randomized controlled trials comparing the management strategies of anterior shoulder instability. Am J Sports Med.

[7] Doll J., Neide A., Mick P., Brunnemer U., Schmidmaier G., Fischer C. (2022). Functional outcome and CEUS-assessed deltoid muscle vitality after fracture-specific versus standard prosthetic design in reverse shoulder arthroplasty for trauma. J Orthop Res.

[8] Doyle T.R., Davey M.S., Hurley E.T. (2022). The statistical fragility of management options for acute achilles tendon ruptures; a systematic review of randomized control trial with fragility analysis. J ISAKOS.

[9] Edwards T.B., Trappey G.J., Riley C., O'Connor D.P., Elkousy H.A., Gartsman G.M. (2012). Inferior tilt of the glenoid component does not decrease scapular notching in reverse shoulder arthroplasty: results of a prospective randomized study. J Shoulder Elbow Surg.

[10] Engel N.M., Holschen M., Schorn D., Witt K.A., Steinbeck J. (2021). Results after primary reverse shoulder arthroplasty with and without subscapularis repair: a prospective-randomized trial. Arch Orthop Trauma Surg.

[11] Evaniew N., Files C., Smith C., Bhandari M., Ghert M., Walsh M. (2015). The fragility of statistically significant findings from randomized trials in spine surgery: a systematic survey. Spine J.

[12] Fackler N.P., Ehlers C.B., Callan K.T., Amirhekmat A., Smith E.J., Parisien R.L. (2022). Statistical fragility of single-row versus double-row anchoring for rotator cuff repair: a systematic review of comparative studies. Orthop J Sports Med.

[13] Feinstein A.R. (1990). The unit fragility index: an additional appraisal of "statistical significance" for a contrast of two proportions. J Clin Epidemiol.

[14] Flatow E.L., Harrison A.K. (2011). A history of reverse total shoulder arthroplasty. Clin Orthop Relat Res.

[15] Fraser A.N., Bjørdal J., Wagle T.M., Karlberg A.C., Lien O.A., Eilertsen L. (2020). Reverse shoulder arthroplasty is superior to plate fixation at 2 years for displaced proximal humeral fractures in the elderly: a multicenter randomized controlled trial. J Bone Joint Surg Am.

[16] Gobezie R., Shishani Y., Lederman E., Denard P.J. (2019). Can a functional difference be detected in reverse arthroplasty with 135° versus 155° prosthesis for the treatment of rotator cuff arthropathy: a prospective randomized study. J Shoulder Elbow Surg.

[17] Graphpad Clinical calculator. https://www.graphpad.com/quickcalcs/contingency1/.

[18] Greiner S., Schmidt C., Herrmann S., Pauly S., Perka C. (2015). Clinical performance of lateralized versus non-lateralized reverse shoulder arthroplasty: a prospective randomized study. J Shoulder Elbow Surg.

[19] Hagen M.S., Allahabadi S., Zhang A.L., Feeley B.T., Grace T., Ma C.B. (2020). A randomized single-blinded trial of early rehabilitation versus immobilization after reverse total shoulder arthroplasty. J Shoulder Elbow Surg.

[20] Jonsson E., Ekholm C., Salomonsson B., Demir Y., Olerud P. (2021). Reverse total shoulder arthroplasty provides better shoulder function than hemiarthroplasty for displaced 3- and 4-part proximal humeral fractures in patients aged 70 years or older: a multicenter randomized controlled trial. J Shoulder Elbow Surg.

[21] Khan M., Evaniew N., Gichuru M., Habib A., Ayeni O.R., Bedi A. (2017). The fragility of statistically significant findings from randomized trials in sports surgery: a systematic survey. Am J Sports Med.

[22] Kuhn J.E. (2010). Levels of evidence and standardizing the reporting of research. J Shoulder Elbow Surg.

[23] Laas N., Engelsma Y., Hagemans F.J.A., Hoelen M.A., van Deurzen D.F.P., Burger B.J. (2021). Reverse or hemi shoulder arthroplasty in proximal humerus fractures: a single-blinded prospective multicenter randomized clinical trial. J Orthop Trauma.

[24] Lopiz Y., Alcobía-Díaz B., Galán-Olleros M., García-Fernández C., Picado A.L., Marco F. (2019). Reverse shoulder arthroplasty versus nonoperative treatment for 3- or 4-part proximal humeral fractures in elderly patients: a prospective randomized controlled trial. J Shoulder Elbow Surg.

[25] Lübbeke A., Rees J.L., Barea C., Combescure C., Carr A.J., Silman A.J. (2017). International variation in shoulder arthroplasty. Acta Orthop.

[26] McCormick K.L., Tedesco L.J., Swindell H.W., Forrester L.A., Jobin C.M., Levine W.N. (2021). Statistical fragility of randomized clinical trials in shoulder arthroplasty. J Shoulder Elbow Surg.

[27] McCulloch P., Taylor I., Sasako M., Lovett B., Griffin D. (2002). Randomised trials in surgery: problems and possible solutions. BMJ.

[28] Navarro R.A., Mellano C.R., Sievers D.A., Harrast J.J., Carpenter J.E., Jackson K.R. (2021). Trends in reverse total shoulder arthroplasty: how the early trends in new innovation provide experience in utilization of later designs. J Orthop Exp Innovat.

[29] Page M.J., McKenzie J.E., Bossuyt P.M., Boutron I., Hoffmann T.C., Mulrow C.D. (2021). The PRISMA 2020 statement: an updated guideline for reporting systematic reviews. BMJ.

[30] Parisien R.L., Ehlers C., Cusano A., Tornetta P., Li X., Wang D. (2021). The statistical fragility of platelet-rich plasma in rotator cuff surgery: a systematic review and meta-analysis. Am J Sports Med.

[31] Parisien R.L., Trofa D.P., Cronin P.K., Dashe J., Curry E.J., Eichinger J.K. (2021). Comparative studies in the shoulder literature lack statistical robustness: a fragility analysis. Arthrosc Sports Med Rehabil.

[32] Parsons N.R., Hiskens R., Price C.L., Achten J., Costa M.L. (2011). A systematic survey of the quality of research reporting in general orthopaedic journals. J Bone Joint Surg Br.

[33] Poon P.C., Chou J., Young S.W., Astley T. (2014). A comparison of concentric and eccentric glenospheres in reverse shoulder arthroplasty: a randomized controlled trial. J Bone Joint Surg Am.

[34] Ruzbarsky J.J., Rauck R.C., Manzi J., Khormaee S., Jivanelli B., Warren R.F. (2019). The fragility of findings of randomized controlled trials in shoulder and elbow surgery. J Shoulder Elbow Surg.

[35] Sebastiá-Forcada E., Cebrián-Gómez R., Lizaur-Utrilla A., Gil-Guillén V. (2014). Reverse shoulder arthroplasty versus hemiarthroplasty for acute proximal humeral fractures. A blinded, randomized, controlled, prospective study. J Shoulder Elbow Surg.

[36] Sexton S., Ferguson N., Pearce C., Ricketts D. (2008). The misuse of ‘no significant difference’ in British orthopaedic literature. Ann R Coll Surg Engl.

[37] Shen Y., Cheng X., Zhang W. (2019). The fragility of randomized controlled trials in intracranial hemorrhage. Neurosurg Rev.

[38] Sterne J.A.C., Savović J., Page M.J., Elbers R.G., Blencowe N.S., Boutron I. (2019). RoB 2: a revised tool for assessing risk of bias in randomised trials. BMJ.

[39] Tignanelli C.J., Napolitano L.M. (2019). The fragility index in randomized clinical trials as a means of optimizing patient care. JAMA Surg.

[40] Torrens C., Amestoy J., Rodríguez-Delourme I., Santana F. (2021). Positioning of the metaglene in reverse shoulder arthroplasty: deltopectoral versus anterosuperior approach: a prospective randomized trial. J Shoulder Elbow Surg.

[41] Torrens C., Guirro P., Miquel J., Santana F. (2016). Influence of glenosphere size on the development of scapular notching: a prospective randomized study. J Shoulder Elbow Surg.

[42] Torrens C., Miquel J., Martínez R., Santana F. (2020). Can small glenospheres with eccentricity reduce scapular notching as effectively as large glenospheres without eccentricity? A prospective randomized study. J Shoulder Elbow Surg.

[43] Van de Kleut M.L., Yuan X., Athwal G.S., Teeter M.G. (2022). Are short press-fit stems comparable to standard-length cemented stems in reverse shoulder arthroplasty? A prospective, randomized clinical trial. J Shoulder Elbow Surg.

[44] Van de Kleut M.L., Yuan X., Teeter M.G., Athwal G.S. (2022). Bony increased-offset reverse shoulder arthroplasty vs. metal augments in reverse shoulder arthroplasty: a prospective, randomized clinical trial with 2-year follow-up. J Shoulder Elbow Surg.

[45] Vara A.D., Koueiter D.M., Pinkas D.E., Gowda A., Wiater B.P., Wiater J.M. (2017). Intravenous tranexamic acid reduces total blood loss in reverse total shoulder arthroplasty: a prospective, double-blinded, randomized, controlled trial. J Shoulder Elbow Surg.

[46] Wagner E.R., Farley K.X., Higgins I., Wilson J.M., Daly C.A., Gottschalk M.B. (2020). The incidence of shoulder arthroplasty: rise and future projections compared with hip and knee arthroplasty. J Shoulder Elbow Surg.

[47] Wasserstein R.L., Lazar N.A. (2016). The ASA statement on p-values: context, process, and purpose. Am Statistician.

[48] Young B.L., Connor P.M., Schiffern S.C., Roberts K.M., Hamid N. (2020). Reverse shoulder arthroplasty with and without latissimus and teres major transfer for patients with combined loss of elevation and external rotation: a prospective, randomized investigation. J Shoulder Elbow Surg.

