# Supplementary material for: The statistical fragility of the management options for reverse shoulder arthroplasty: a systematic review of randomized control trial with fragility analysis
Source: JSES Rev Rep Tech. 2023 Apr 7;3(3):279–84. doi: 10.1016/j.xrrt.2023.03.002 (PMC10426549; doi:10.1016/j.xrrt.2023.03.002)
Supplement: Supplementary Appendix S1 [file mmc1.docx]

**APPENDIX**

| **Study ID** |  | **D1** | **D2** | **D3** | **D4** | **D5** | **Overall** |
| --- | --- | --- | --- | --- | --- | --- | --- |
| Van de Kleut 2022-1 | |  |  |  |  |  |  |
| Van De Kleut 2022-2 | |  |  |  |  |  |  |
| Torrens 2021 | |  |  |  |  |  |  |
| Torrens 2020 | |  |  |  |  |  |  |
| Torrens 2016 | |  |  |  |  |  |  |
| Young 2020 | |  |  |  |  |  |  |
| Lopiz 2019 | |  |  |  |  |  |  |
| Gobeize 2019 | |  |  |  |  |  |  |
| Frazer 2020 | |  |  |  |  |  |  |
| Greiner 2015 | |  |  |  |  |  |  |
| Sebastiá-Forcada 2011 | |  |  |  |  |  |  |
| Poon 2014 | |  |  |  |  |  |  |
| Edwards 2012 | |  |  |  |  |  |  |
| Vara 2017 | |  |  |  |  |  |  |
| Doll 2022 | |  |  |  |  |  |  |
| Laas 2016 | |  |  |  |  |  |  |
| Jonsson 2020 | |  |  |  |  |  |  |
| Hagen 2020 | |  |  |  |  |  |  |
| Engel 2019 | |  |  |  |  |  |  |
|  |  |  |  |  |  |  |  |
| \|  \| \| --- \| | Low risk | D1 | Randomization process | | | |  |
| \|  \| \| --- \| | Some concerns | D2 | Deviations from the intended interventions | | | | |
| \|  \| \| --- \| | High risk | D3 | Missing outcome data | | | |  |
|  |  | D4 | Measurement of the outcome | | | | |
|  |  | D5 | Selection of the reported result | | | | |
